# An Augmented Reality-Based Concept for Anchor-Free Assessment of Tremor-Related Hand Movement Patterns

**DOI:** 10.3390/jimaging12090421

**Published:** 2026-09-07

**Authors:** Volodymyr Hrytsyk, Maksym Kochut, Uliana Marikutsa, Oleh Lytovchenko, Oleh Berezyuk, Vitalii Hrendus, Mariia Nazarkevych

**Affiliations:** 1 Institute of Computer Science and Information Technologies, Lviv Polytechnic National University, 79013 Lviv, Ukraine; maksym.kochut@gmail.com (M.K.); uliana.b.marikutsa@lpnu.ua (U.M.); oleh.v.lytovchenko@lpnu.ua (O.L.); 2 Department of Psychiatry and Psychotherapy, Danylo Halytsky Lviv National Medical University, 79010 Lviv, Ukraine; sssriii@i.ua; 3 St. Panteleimon Hospital of the First Territorial Medical Association of Lviv, 79059 Lviv, Ukraine; grendusvitalik17@gmail.com

**Keywords:** augmented reality, medical imaging, computer-aided screening, computer vision, neurodegeneration, screening, tremor screening, Parkinson’s disease

## Abstract

Neurological disorders are a major cause of disability and mortality worldwide, and accessible methods for detecting tremor-related trajectory deviations remain an important research challenge. We present an augmented reality (AR)-based approach for evaluating trajectory deviations associated with tremor-related hand movements in Parkinson’s disease (PD) and neurological sequelae of traumatic brain injury (TBI). Unlike conventional surface-based drawing tests, the proposed approach removes the physical support point that may facilitate compensatory stabilization of hand movements during task performance. The method implementing this concept was preliminarily evaluated in a pilot cohort of 131 participants, including 50 healthy controls, 6 patients with PD, and 75 combat veterans with TBI, and was compared with a standard geometric figure drawing test. In the PD subgroup, the AR protocol flagged algorithmically detected trajectory deviations in all six participants. Given the small PD sample and the limited number of additional findings in the TBI group, these observations should be interpreted as preliminary and hypothesis-generating rather than as evidence of proof-of-concept accuracy or sensitivity. The results suggest that the anchor-free AR-based trajectory-assessment method may warrant further investigation as a potential approach for tremor-related movement analysis. Larger clinically validated studies with independent confirmation and standard concept metrics will be required to determine the clinical utility of the proposed method.

## 1. Introduction

Neurological disorders are among the leading causes of disability worldwide. Among movement disorders, Parkinson’s disease (PD) is one of the most clinically important conditions because tremor, bradykinesia, and impaired motor control can often be detected through quantitative analysis of hand movements. In addition, neurological sequelae of traumatic brain injury (TBI), particularly in combat veterans, may include tremor-related abnormal movements and impaired upper-limb motor coordination. Therefore, accessible methods for detecting abnormal hand-movement patterns are of considerable interest for preliminary screening and remote neurological assessment.

This paper explores a method for algorithmic assessment of hand-movement trajectories associated with tremor-related neurological disorders [1].

Alzheimer’s disease is a type of dementia that affects about 6% (one in sixteen) of people over the age of 65 [2]. In this context, the diagnosis focuses on the determined parameters of the patient’s abnormal movements (T-object) caused by the negative effects of a specific set of neural nodes. In the USA, the National Institute of Neurological Disorders and Stroke (NINDS) and the Alzheimer’s Association have compiled the most widely used set of criteria for diagnosing Alzheimer’s [3].

According to the criteria, to make a clinical diagnosis of possible neurological disease, cognitive impairment must be confirmed.

1. Most often, neurological disorders affect eight domains (in various combinations): Memory, speech skills, perception of the environment, constructive abilities, orientation in space and time, personality, problem-solving skills, functioning, and self-sufficiency. These domains are equivalent to the NINCDS-ADRDA criteria [4]. Tremor, characterized by involuntary shaking of a body part, is the most common movement disorder [5]. The domain is most effective when capturing the moment of tremor appearance as a marker of the onset of negative processes. Establishing the state of the “early/first stage” enables preventive actions to begin before the disease progresses further.

This work focuses on the early detection of disease signs, so we will consider the early stage in more detail. Progressive memory decline [6] and agnosia (disorder of recognition processes) in neurodegenerative diseases, sooner or later, lead to confirmation of the diagnosis. In a small number of patients, it is not memory disorders that come to the fore, but disorders of speech, executive functions, perception, or movement disorders (apraxia (including tremor)) [7]. When drawing, writing, performing self-care activities in the kitchen, putting on clothes, and performing other tasks that require fine motor skills, a person may seem clumsy due to problems with coordination and movement planning [8]. As the disease progresses, a person is often quite capable of performing many tasks independently but may require assistance or care when attempting manipulations that require special cognitive effort, as discussed by Förstl H and Kurz A [7]. Among other effects, Parkinson’s disease impairs motor control, primarily of the upper and lower limbs, and causes tremors and muscle stiffness [9].

Researchers worldwide are seeking convenient and effective screening methods. To date, there have been developments in computational models of movements, for example, walking [10] or precision grip [11]. Eye movements have been studied [12,13]. Eye behavior is considered an informative feature [14]. Therefore, in IT, it is relevant to distinguish characteristic features, in particular, area/lines [15] or area/curves [16]. Mathematical solutions for automating the diagnostic process are being studied [17].

**Figure 1:**
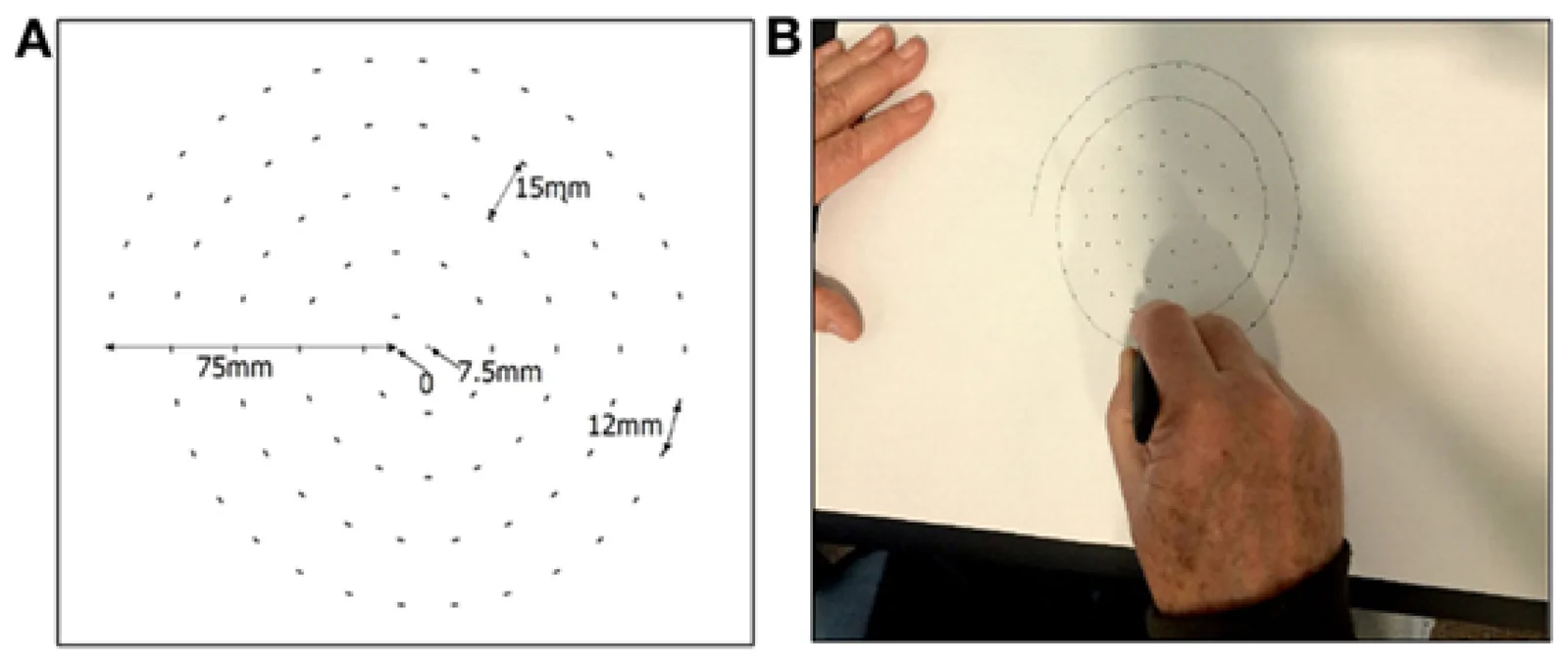
The spiral view (**A**) and the process of passing the test (**B**).

Thus, at the first (simplest) stage, patients’ reflexes, ability to get up from a chair and walk around the room, and muscle tone and strength are assessed. During the neurological examination, vision, hearing, coordination, and balance are assessed. The first symptoms are often mistaken for signs of aging or a reaction to stress, as discussed by K. Palmer and A. K. Berger [18]. Examining available diagnostic methods [19,20,21] and state assessment methods [22,23,24], the authors developed a method for detecting upper-limb tremor as a basic element of early dementia diagnosis [25]. In 2017, Australian researchers from RMIT University conducted a study investigating the impact of Parkinson’s disease on people’s movements. A total of 55 people took part in the study (27 of them had the disease, 28 did not). Each of the participants was asked to draw a spiral on the tablet along the given control points (see Figure 1) [25].

During the test, a tablet with special software measured the speed of movement and the pressure of the pen on the tablet, allowing researchers to distinguish between those with and without the disease and the severity of their condition. The researchers combined speed and pen pressure data into a single measurement for each participant and compared the measurements.

**Figure 2:**
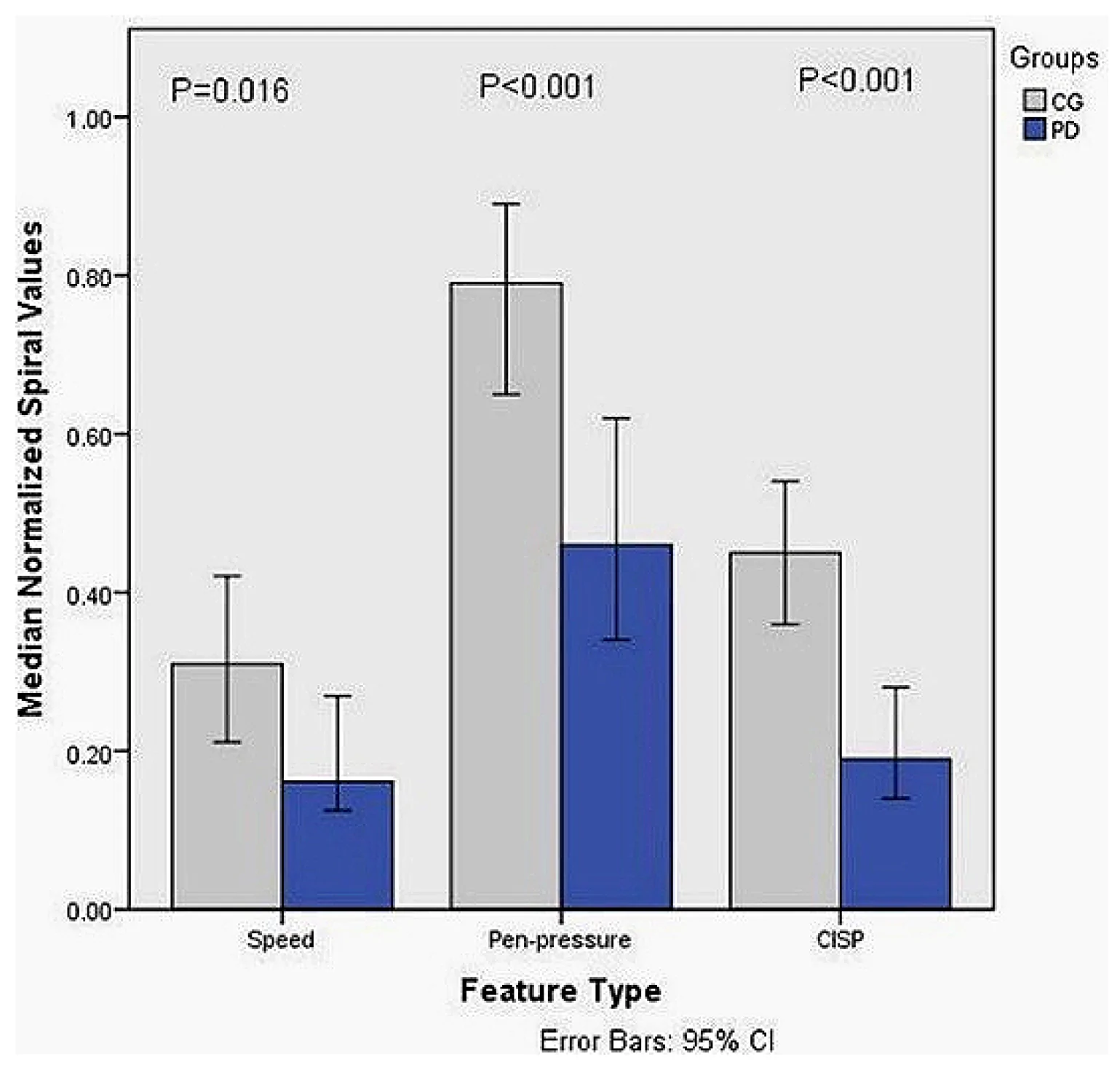
Comparative graph of indicators for healthy controls (CG) and persons with disease (PD).

In the study, the authors concluded that there was a significant difference in indicators between persons with the disease and healthy persons (see Figure 2) [25].

As can be seen from the figure (see Figure 2), such a significant difference in indicators allows us to make a preliminary assessment of the condition of the patient who underwent testing. David Dexter, deputy director of Parkinson’s Research UK, confirmed this finding: “Current tests for the disease cannot accurately measure how advanced a person’s condition is. This may affect the ability to select the right people for clinical trials, which is important for developing new and better treatments for Parkinson’s disease.” [26].

The authors of the article Alty J., Cosgrove J., Thorpe D. [27] believe that people with Parkinson’s disease often write and draw slowly, producing small handwriting and spirals formed from tight turns. The reduced size and speed of hand movements reflect bradykinesia, the main motor symptom of Parkinson’s disease. The authors believe that spiral drawings are more sensitive than small handwriting, especially when small handwriting has been present throughout life.

Action tremor is present during drawing in almost half of patients with Parkinson’s disease, and most importantly, the reduced diameter and increased density of turns distinguish Parkinsonian tremor with sufficient specificity.

Therefore, by analyzing the speed and nature of a person’s drawing of the Archimedean spiral, it may be possible to identify trajectory patterns potentially associated with tremor-related motor impairment in Alzheimer’s or Parkinson’s disease, particularly tremor and reduced movement speed.

In the study by Megha Kamble, Prashant Shrivastava, and Megha Jain, this topic was investigated using various methods and patient samples [28]. Recent studies have moved beyond conventional pen-and-paper spiral tests by digitizing hand-movement trajectories, thereby reducing some limitations of purely surface-based visual assessment [1]. Smartphone-based finger drawing enables the direct acquisition of finger trajectories during spiral execution [29]; however, because the finger still moves on a screen surface, this approach retains a physical support point that may act as a mechanical anchor and support compensatory stabilization of movement abnormalities. Wearable-sensor-based aerial spiral analysis addresses the anchor problem by analyzing unsupported hand movements [30]; however, it requires additional body-mounted instrumentation.

The proposed AR-based concept combines the main advantages of these two approaches: it eliminates the mechanical anchor-based compensatory mechanism while avoiding the use of wearable sensors. Specifically, it implements an AR-guided visual task with camera-based hand-position acquisition as an anchor-free screening component for screening tremor-related abnormal movements. This combination enables preliminary remote assessment using commonly available camera-equipped devices, such as smartphones or personal computers, without specialized clinical equipment. As a result, the approach may help reduce unnecessary preliminary visits to medical institutions, patient stress, and the initial screening burden on the healthcare system. Also, we combine our AR-based approach with a CNN-based perception method [31]. When selecting a complexity measure assessment, ref. [32] was analyzed; the “problem of counting cycles” was analyzed as well. A modern approach to the state assessment strategy was studied. Changes in node connectivity, not just edge changes, are studied, affecting measurements and ways of working with open neuroimaging databases.

This study aims to preliminarily evaluate the viability of an augmented-reality-based concept that eliminates the mechanical anchor-based compensatory mechanism while avoiding the use of wearable sensors as a method for algorithmic assessment of trajectory deviations in participants with tremor-related movement abnormalities. This study also aims to investigate the performance of the method implementing this concept in a pilot screening study.

The diagnostic procedure is performed in the augmented-reality mode. Thus, the available data, from which conclusions will be drawn, are the patient’s hand position *(x, y)* and time *(t)*. Reaction time is an important indicator [4]. Additionally, the ability to perceive objects (such as spirals) and to coordinate one’s movements with respect to virtual 3D objects is implicitly evaluated. At this stage of research, the object is a 2D planar spiral positioned in 3D space.

To summarize, the main contributions of this work are as follows:

1. Unlike existing classical surface-based approaches, the proposed concept in an augmented-reality environment eliminates the mechanical “anchor”—a physical point of support for the limb. This reduces the possibility of using compensatory stabilization mechanisms that can mask tremor symptoms during drawing on solid surfaces, particularly in the early stages of the disease. Compared with wearable-sensor approaches, an additional advantage of the proposed method is its potential for large-scale use because it does not require body-mounted sensors or specialized equipment beyond commonly available camera-equipped devices. Consequently, the AR-based concept increases the potential for detecting trajectory deviations associated with neuromotor disorders that may be less visible during classical surface-based tests. This study preliminarily evaluates the viability of this AR-based approach.
2. The concept enables the development of a remote screening system, reducing the need for patients to visit medical institutions for preliminary assessment or undergo more complex diagnostic procedures. Its potential advantages include reducing patient stress, lowering the burden on the healthcare system, and saving time spent on in-person visits. By using standard hardware, such as smartphone cameras or PC webcams, the proposed method enables primary screening with widely available consumer devices. The procedure is cost-effective and accessible. Thus, the technology being studied will serve as a practical component of a future diagnostic workflow, including use at the family medicine level for screening during remote consultations or for self-monitoring. From an algorithmic perspective, the practicality of the proposed method is supported by its computational simplicity: the control points of the virtual spiral are predefined, and the trajectory-performance score is updated frame by frame using Euclidean distance calculations and two deviation-registration conditions. Therefore, the computational complexity is linear in the number of recorded trajectory points, enabling the procedure to run in real time on conventional consumer hardware.

The remainder of this paper is organized as follows. Section 2 describes the proposed method for screening abnormal movements using augmented reality and explains the analytical solution and the mathematical conditions for deviation registration. The results of the experimental studies and an analysis of how different constants influence the trajectory-performance score are provided in Section 3. Section 4 visualizes the evaluation of the recognition process. Section 5 is dedicated to discussing the findings, specifically the analysis of the “anchor” effect and the details of testing across various patient categories. Section 6’s “Limitations” show that the obtained results should be interpreted as preliminary evidence of technical feasibility and hypothesis-generating observations rather than as evidence of diagnostic accuracy. Section 7 presents the experiment planned as future work. Section 8 presents the most important conclusions of the work.

## 2. Materials and Methods

The research is based on the method for classifying states by identifying atypical movements (caused by cognitive factors) using interactions with augmented reality and spectral analysis in heterogeneous environments. An approach to constructing hybrid models that describe the state and behavior of abnormal neurological movements (ANM) in specific body parts of a T-object under the cognitive influence of a specific neural node group is proposed.

The trajectory-performance score is an algorithmic measure of geometric deviation from the reference spiral trajectory. It quantifies how accurately the participant follows the predefined path under the proposed scoring rules. The score should not be interpreted as a direct measure of clinical tremor severity, Parkinson’s disease severity, or overall neurological impairment. Clinical severity assessment requires an independent neurological evaluation and standardized tremor rating scales, which were not incorporated into the present pilot feasibility study.

### 2.1. Generation of the Reference Archimedean Spiral

The virtual reference trajectory was defined as an Archimedean spiral. In polar coordinates, the spiral is described by

(1) r(θ)=a+bθ, 0 ≤ θ ≤ θmax,

where a is the initial radius, *b* is the radial growth coefficient that determines the spacing between successive turns, and *θ* is the polar angle.

For the AR implementation, the spiral was sampled by a sequence of discrete angular values:

(2) $$\theta_{i}\;=\;i\cdot \Delta \theta ,\;\;i\;=\;0,1,\ldots ,N-1,$$

where *Δθ* = $\theta_{max}$/(*N*−1), and *N* is the number of control points. The value of $\theta_{max}$ was selected to produce the required number of spiral turns within the predefined interaction area, while *Δθ* was chosen to ensure uniform sampling of the reference trajectory.

For each angular value $\theta_{i}$, the corresponding radius was computed as

(3) $$r_{i}=a+b\cdot \theta_{i}.\;$$

The polar coordinates were then converted into Cartesian coordinates according to

(4) $$x_{i}=r_{i}\;cos\;\theta_{i},$$

(5) $$y_{i}=r_{i}\;sin\;\theta_{i}.\;\;$$

The resulting set of points,

(6) $$C_{i}=\left(x_{i},y_{i}\right),\;\;\;\;\;\;\;\;i=0,1,\ldots ,N-1,\;$$

formed the reference spiral used by the scoring algorithm. These points represent the invisible control points described in the experimental protocol. During testing, the participant moved their fingertip along the visible spiral trajectory, while deviations were evaluated relative to the next control point on the reference curve.

To ensure consistent testing conditions, the parameters *a*, *b*, $\theta_{max}$, and *N* were fixed for all participants. The generated Cartesian coordinates were rendered in the AR coordinate system after linear scaling and translation to fit the predefined interaction area.

This formulation provides a deterministic mathematical definition of the reference trajectory and allows exact reproduction of the control-point generation procedure used in the proposed AR-based assessment method.

Participants performed the task from the center toward the outer end of the spiral (inside-out).

### 2.2. Analytical Solution

As mentioned in the paragraph “Statement of the problem”, in the context of augmented reality, the available data, on which conclusions will be drawn, are the patient’s hand position *(x, y)* and time (*t*). In addition, the augmented reality environment enables the creation of auxiliary virtual objects to assess the patient’s condition. Building on the previously discussed work of A. P. Legrand and I. Rivals [19], control points will be used to analyze the patient’s condition in augmented reality. Control points are evenly spaced, invisible checkpoints along the spiral’s trajectory. The patient must swipe their finger over the trajectory. The advantage of this method is that the patient does not need to know exactly where these points are, and the method makes it possible to assess the patient’s movements.

**Figure 3:**
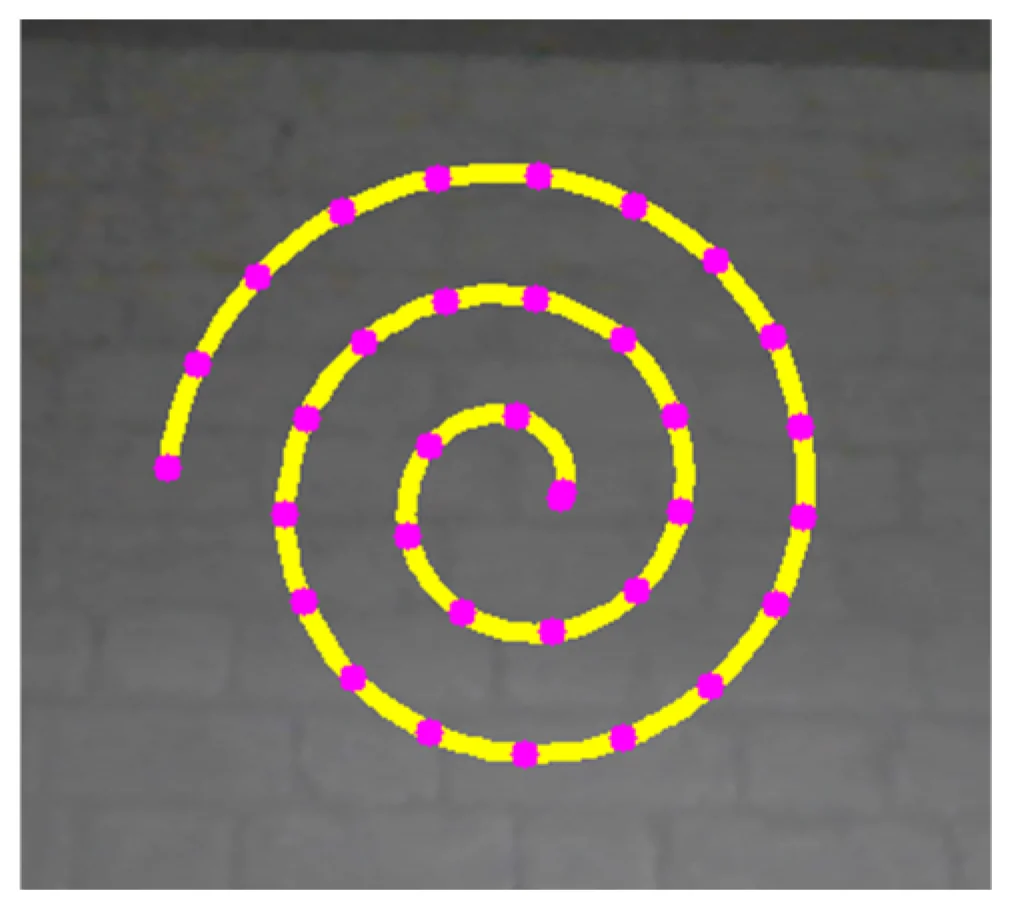
View of the spiral with control points applied to it.

Figure 3 shows (in yellow) the trajectory along which the patient should swipe their finger and (in pink) the control points used to analyze the patient’s movements.

For the analysis to be as accurate as possible, during testing, the patient will not see the control points; ideally, he should not be aware of their existence at all; therefore, he can focus as much as possible on his main task—moving his finger along the spiral’s trajectory. Having introduced the concept of control points and their coordinates, we can proceed to the description of the analytical solution to the problem of algorithmic assessment of the participant’s movement trajectory based on the trajectory of his finger movements along a spiral.

Let

x, y be the coordinates of the finger at the moment t.

$$c_{x},c_{y}\;be\;the\;coordinates\;of\;the\;next\;control\;point.$$

$${c_{prev}}_{x},{c_{prev}}_{y}\;be\;the\;coordinates\;of\;the\;previous\;control\;point.$$

$$dist_{t}\;be\;the\;distance\;to\;the\;control\;point\;at\;the\;moment\;t.$$

$$dis{t_{prev}}_{t}\;be\;the\;distance\;to\;the\;previous\;control\;point\;at\;the\;moment\;t.$$

$$dist_{c}\;be\;the\;distance\;from\;the\;previous\;control\;point\;to\;the\;next.$$

Then, all *dist* distance formulas are calculated using the Euclidean distance formula:

(7) $$dist_{t}=\sqrt{{\left(x-c_{x}\right)}^{2}+{\left(y-c_{y}\right)}^{2}}$$

(8) $$dist_{prev_{t}}=\sqrt{{\left(x-{c_{prev}}_{x}\right)}^{2}+{\left(y-{c_{prev}}_{y}\right)}^{2}}$$

(9) $$dist_{c}=\sqrt{{\left({c_{prev}}_{x}-c_{x}\right)}^{2}+{\left({c_{prev}}_{y}-c_{y}\right)}^{2}}$$

At the beginning of the test, the algorithmic trajectory-performance score is set to 100. This score quantifies how closely the participant follows the target trajectory according to the proposed geometric scoring rules. During testing, deviations from the trajectory accumulate penalties that reduce the score. A score of 100 corresponds to an ideal trajectory following without accumulated penalties under the proposed algorithm, whereas lower values indicate greater accumulated deviation. At this stage, the score is used as an algorithmic performance index; clinical calibration against UPDRS or other tremor-severity scales will require larger clinically annotated validation cohorts.

### 2.3. Conditions for Registration of Deviation from the Trajectory

The first condition:

(10) $$dist_{t}>dist_{t-1}\cdot {const}_{1}$$

(11) $${const}_{1}\geq 1$$

where *const*_1_ is a constant that determines the difficulty of passing the test. The smaller it is, the more difficult it is to pass the test. That is, it regulates the degree of tolerance to the patient’s mistakes and how often the patient is penalized. If *const*_1_ *=* 1, the penalty is calculated for the minimum deviation from the trajectory.

The amount of the penalty is calculated according to the formula:

(12) $$\Delta d\;=\;max(0,\;{dist}_{t+1}\;-\;{dist}_{t})$$

(13) $${p_{const}}_{1}>0.$$

Here, ${p_{const}}_{1}$ is the penalty level constant. The greater its value, the greater the penalty. That is, it sets the amount of the penalty for a patient’s mistake.

To avoid ambiguity, the distance measures used in the algorithm are defined as Euclidean distances. The penalty is applied only when the participant moves farther away from the next control point than at the previous time step. Accordingly, the penalty is computed from the non-negative increase in distance.

This formulation guarantees that penalties are always non-negative and that the trajectory-performance score cannot increase as a result of a penalty calculation. If the participant moves closer to the target trajectory, no penalty is applied.

It follows from formula (5) that the penalty depends on the change in distance to the next control point between consecutive time intervals. If the participant moves farther away from the reference trajectory, the accumulated penalty increases and the trajectory-performance score decreases. Thus, the algorithm provides a simple geometric measure of deviation from the target path. Larger accumulated deviations may be consistent with trajectory-deviation patterns, whereas quantification of clinical tremor severity will require further validation.

### 2.4. Control-Point Progression Rule

The reference spiral consists of an ordered sequence of control points, $C_{1}$, $C_{2}$, …, $C_{n}$. At any time step, the algorithm tracks the current target control point $C_{k}$. The target remains *C_k_* until the fingertip enters a circular acceptance region centered at $C_{k}$ with radius *r__accept_*. When the Euclidean distance satisfies

(14) $$\left|\left|P_{t}-C_{k}\right|\right|\leq \;r_{accept},$$

the control point is considered reached, and the algorithm advances to the next control point $C_{k+1}$. If the acceptance condition is not satisfied, all deviation calculations continue to be performed relative to the current target control point $C_{k}$.

The acceptance radius $r_{accept}$ was fixed for all participants and set to be smaller than the spacing between adjacent control points to prevent premature advancement.

### 2.5. Algorithm-Positive Classification Criterion

After processing the complete trajectory, the algorithm produces a final trajectory-performance score in the range from 0 to 100, where higher scores indicate better trajectory performance.

A trajectory was classified as algorithm-positive when the final trajectory-performance score satisfied

*score* < *T*,

where *T* = 70 was the predefined decision threshold used throughout the study.

Trajectories with

*score* ≥ *70*

were classified as algorithm-negative.

The threshold T was fixed before data analysis and applied identically to all participants and experimental conditions. In the present pilot study, the threshold was selected as a predefined algorithmic decision parameter intended to separate trajectories with substantial cumulative deviations from those with comparatively minor deviations. The threshold should not be interpreted as a clinically validated diagnostic cut-off and requires calibration and validation against an independent clinical reference standard in future studies.

### 2.6. Definition of the Trajectory Deviation Measure

The second deviation component quantifies the geometric deviation of the fingertip from the local spiral trajectory. Let the current target control point be $C_{k}$ and the next control point be $C_{k+1}$. The local reference trajectory is represented by the line segment connecting these two control points.

For the fingertip position $P_{t}$, the trajectory deviation measure is defined as the minimum Euclidean distance from $P_{t}$ to the line segment [$C_{k}$, $C_{k+1}$]:

(15) $$eviation\_measure\;=\;min\_\{Q\;in\;[C_{k},\;C_{k+1}]\}\;||P_{t}\;-\;Q||,$$

where Q is any point on the segment connecting $C_{k}$ and $C_{k+1}$, and ||·|| denotes the Euclidean norm.

The reference measure is defined as the length of the current spiral segment:

(16) $${reference}_{measure}\;=\;||C_{k+1}\;-\;C_{k}||.$$

The second deviation condition is satisfied when

(17) $${deviation}_{measure}>{const}_{2}\times {reference}_{measure}$$

When this condition is satisfied, the corresponding penalty is computed as

(18) $${penalty}_{2}=p_{const2}\times {deviation}_{measure}.$$

### 2.7. Integrated Trajectory-Performance Scoring Algorithm

The algorithm (show Algorithm 1) advances through the control points sequentially.
**Algorithm 1.** Integrated trajectory-performance scoring algorithm      Input: Ordered control points $C_{1}$, $C_{2}$, …, $C_{n}$ Recorded fingertip positions $P_{1}$, $P_{2}$, …, $P_{m}$ Parameters ${const}_{1}$, ${const}_{2}$ Acceptance radius $r_{accept}$ Skip penalty $p_{skip}$            Initialize: score = 100 k = 1      For t = 1 to m − 1:      Control-point progression      Repeat:               $d_{current}$= ||$P_{t+1}-\;C_{k}$||               If $d_{current}$ ≤ $r_{accept}$:               k = min(k + 1, n)      Else if k < n and ||$P_{t+1}-\;C_{k+1}$|| + $r_{accept}$ < $d_{current}$: score = score − $p_{skip}$ k = k + 1      Else:               Break      Until k = n      Distances to the current target control point      d_t = ||$P_{t}-\;C_{k}$||      d_t+1 = ||$P_{t+1}-\;C_{k}$||       Initialize penalty      penalty = 0       First deviation component      If $d_{t+1}$ > ${const}_{1}$ × $d_{t}$:      penalty = penalty + $p_{const1}$ × ($d_{t+1}-\;d_{t}$)       Second deviation component      Compute the minimum distance from $P_{t+1}$ to the current spiral segment:      ${deviation}_{measure}$ = min_{Q in [$C_{k}$, $C_{k+1}$]} ||$P_{t+1}$ − Q||      ${reference}_{measure}$ = ||$C_{k+1}$ − $C_{k}$||      If ${deviation}_{measure}$ > ${const}_{2}$ ${reference}_{measure}$:         penalty = penalty + $p_{{const}_{2}}$ × ${deviation}_{measure}$      Update score      score = score − penalty      End For      Return      If score < T:         classification = algorithm-positive      Else:         classification = algorithm-negative      Return score, classification

**Figure 4:**
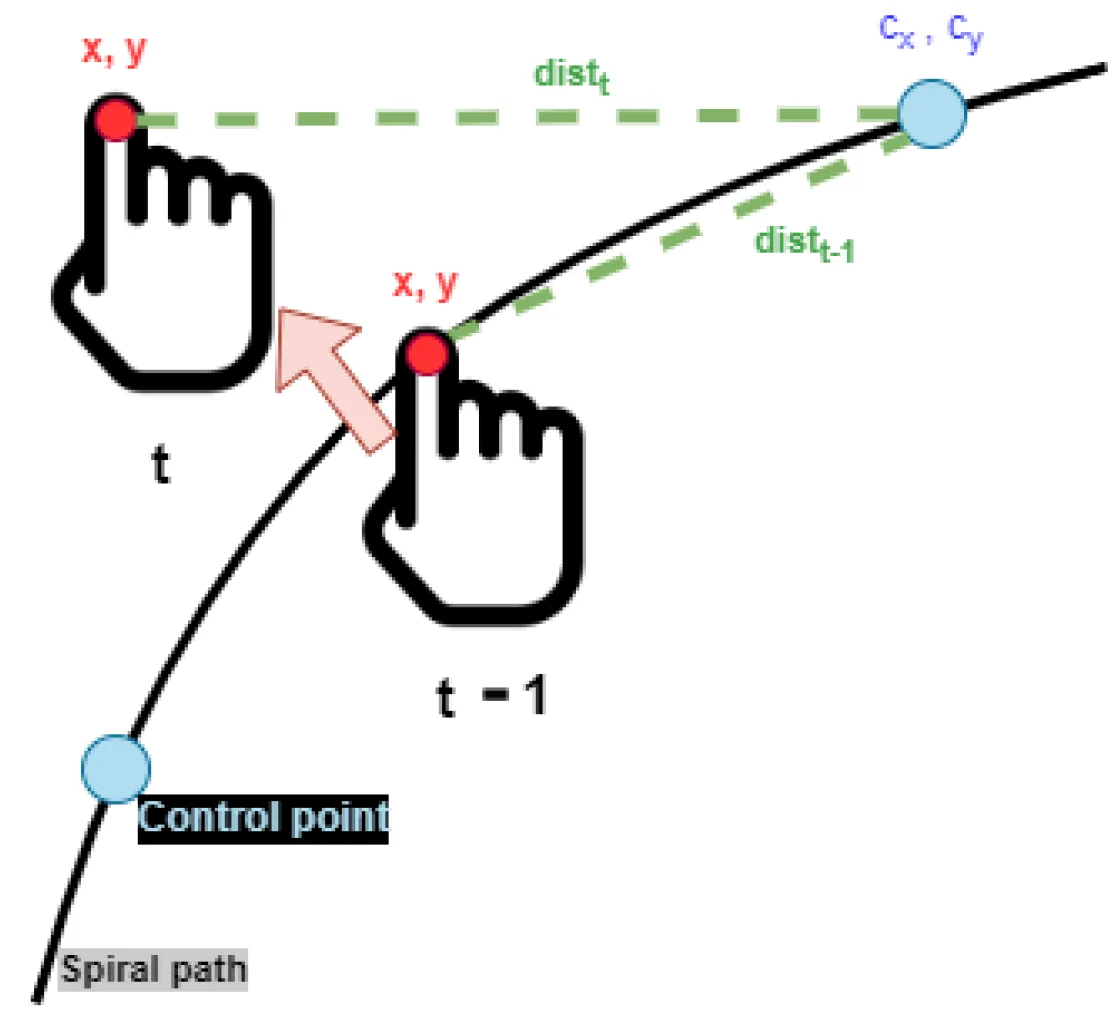
Schematic representation of the operation of the first condition algorithm.

**Figure 5:**
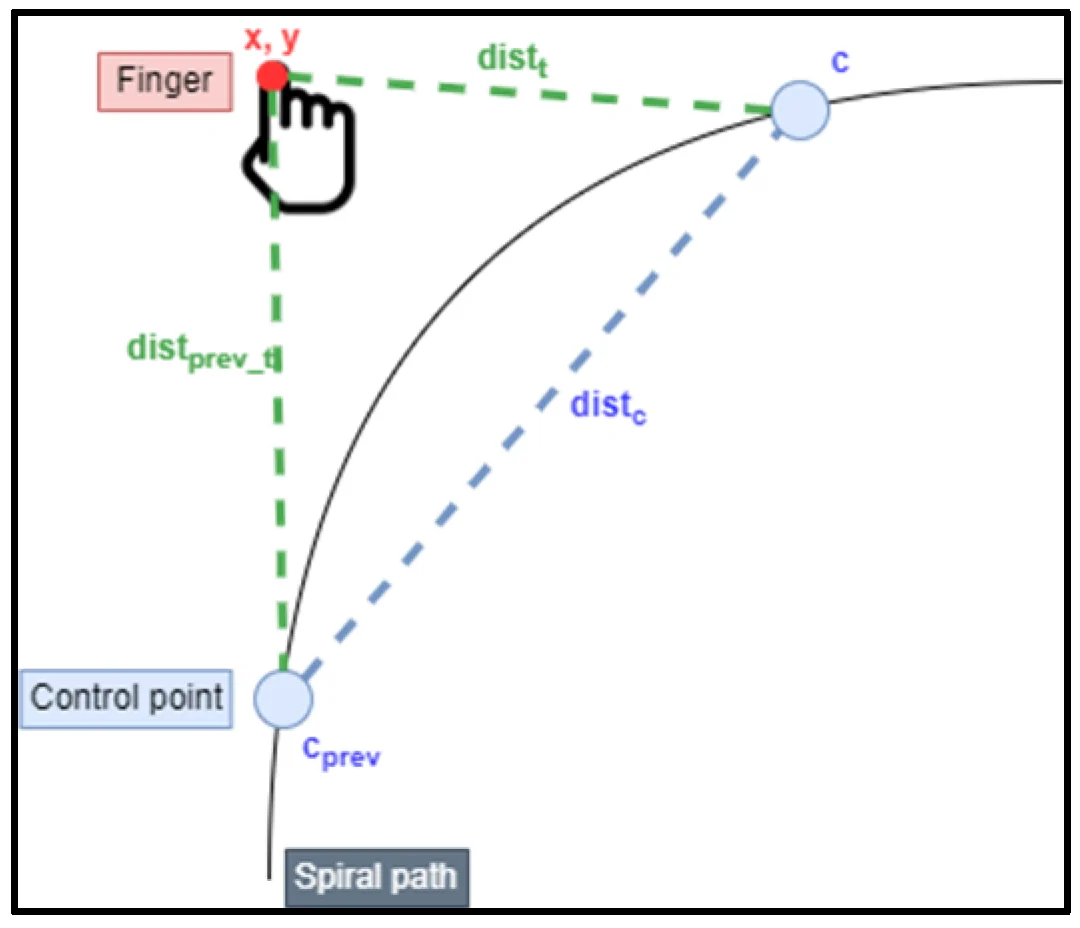
Schematic representation of the operation of the second condition algorithm.

If the fingertip enters the acceptance region of the current control point, that point is considered reached (see Figure 4). If the fingertip moves beyond the current control point and becomes substantially closer to the next control point than to the current one, the current point is considered skipped or overshot. In this case, the algorithm advances to the next control point and applies a fixed skip penalty $p_{skip}$ to prevent artificially avoiding difficult trajectory segments (see Figure 5).

### 2.8. Parameter Selection

The algorithm uses five fixed parameters: the distance-growth threshold ($const_{1}$), the trajectory-deviation threshold ($const_{2}$), the penalty coefficients ($p_{const1}$ and $p_{const2}$), and the algorithm-positive classification threshold (*T*).

These parameters were determined during a preliminary methodological development phase using pilot trajectories acquired outside the study cohort and were fixed before analysis of the participant data reported in this study. The parameters were not optimized using the Parkinson’s disease, traumatic brain injury, healthy control, or pediatric participant data presented in this Section.

The purpose of the preliminary parameter selection was to obtain stable trajectory-performance behavior across repeated test trajectories and to avoid excessive sensitivity to small positional fluctuations. Because the present study was designed as a pilot feasibility investigation, no data-driven optimization, cross-validation, receiver operating characteristic optimization, or post hoc threshold adjustment was performed on the study cohort.

Consequently, the reported algorithm-positive classifications represent the output of a fixed scoring algorithm rather than a model optimized for the current participant sample. The fixed parameter values used throughout the study were ${const}_{1}$ = 1.0, $p_{const1}$ = 0.7, ${const}_{2}$ = 1.1, $p_{const2}$ = 0.1, $r_{accept}$ = 0.015, $p_{skip}$ = 2.0, and *T* = 70. These values remained unchanged for all participants and both experimental conditions.

### 2.9. Algorithm-Positive Trajectory Deviation Classification

The AR-based analysis produced a continuous trajectory-performance score ranging from 0 to 100, where higher scores indicated better agreement between the participant’s trajectory and the reference Archimedean spiral.

A trajectory was classified as algorithm-positive for trajectory deviation when the final trajectory-performance score was below the predefined threshold *T* = 70. Trajectories with *scores* ≥ 70 were classified as algorithm-negative.

The threshold *T* = 70 was established during the methodological development phase using pilot trajectories collected outside the study cohort and was fixed before analysis of the participant data reported in this study. The threshold was not optimized using the healthy control, Parkinson’s disease, traumatic brain injury, or pediatric participant data, and no receiver operating characteristic optimization, cross-validation, or post hoc threshold adjustment was performed on the study cohort.

The same threshold value was applied to all participant groups and to both experimental conditions.

The AR classification was fully automated. The trajectory-performance score and the resulting algorithm-positive or algorithm-negative classification were generated directly by the trajectory-scoring algorithm without visual inspection, manual adjustment, or clinician intervention.

The standard geometric drawing test was evaluated independently. Standard-test positivity was determined using the same trajectory-scoring framework and the same predefined threshold *T* = 70, applied to the trajectory acquired during the standard surface-based drawing condition. Thus, the standard test served as a comparison condition and was not used to define AR abnormality.

The present study did not establish a clinically validated definition of abnormal movement. Algorithm-positive classifications represent detections of substantial trajectory deviations under the proposed scoring algorithm and should not be interpreted as clinically confirmed pathological movement abnormalities. The threshold T should therefore be regarded as a predefined algorithmic decision parameter requiring future validation against an independent clinical reference standard.

### 2.10. Experimental Protocol

The experimental protocol was designed to evaluate the feasibility of the proposed AR-based trajectory assessment under standardized conditions.

**Camera setup.** Hand movements were recorded using a standard integrated RGB camera from a consumer-grade laptop (2019 hardware generation). The camera operated in automatic exposure mode under indoor lighting conditions. Direct sunlight and strong backlighting were avoided because these conditions reduced hand-tracking stability.

**Hand-tracking method.** Finger positions were obtained using a vision-based hand-tracking algorithm implemented with a standard deep-learning hand landmark detection library. The fingertip coordinates were extracted frame by frame and projected onto the virtual spiral coordinate system.

**Sampling rate.** Finger coordinates were recorded at the native frame rate of the camera (approximately 30 frames/s).

**Participant position.** Participants were seated approximately 50–70 cm from the camera. The tested hand was positioned in front of the camera within the predefined interaction area displayed by the software. Participants were instructed to keep their torso relatively stable and to perform the task primarily with upper-limb movement.

**Spiral geometry.** The target trajectory consisted of a planar Archimedean spiral rendered in augmented reality. The spiral was displayed in a fixed orientation and scale for all participants. The virtual spiral occupied the central region of the interaction area and was positioned perpendicular to the camera viewing direction.

**Task procedure.** Participants were instructed to move their index finger along the visible spiral trajectory from the center toward the outer end. Before the recorded measurement, each participant completed one familiarization (practice) trial to become accustomed to the AR interaction. The analysis was performed on the first recorded trial after familiarization.

**Control points.** The spiral trajectory was sampled by uniformly distributed invisible control points placed along the reference curve. The number and spacing of the control points were fixed for all participants. Deviations were evaluated relative to the current target control point, which advanced sequentially according to the control-point progression rule defined above.

**Tracking errors.** Frames in which the fingertip could not be reliably detected by the hand-tracking algorithm were excluded from penalty computation. Short tracking interruptions were ignored, whereas prolonged tracking loss resulted in repetition of the trial.

**Abnormal movement detection.** The current pilot study used the algorithmic trajectory-performance score as a geometric deviation measure. Participants were classified as demonstrating a trajectory deviation pattern when the AR trajectory exhibited substantial deviations from the reference trajectory compared with the behavior observed during the standard geometric drawing test. Because the study lacked an independent clinical reference standard, this classification should be interpreted as an algorithmic detection result rather than a clinically validated diagnosis.

**Blinding.** The trajectory-processing algorithm operated automatically and was independent of participant group assignment. Trajectory-performance scores and algorithm-positive findings were generated directly by the predefined algorithm without visual assessment, manual scoring, or clinician judgment. Investigators responsible for trajectory processing and score computation were blinded to participant group assignments during data analysis. Because the trajectory assessment was algorithm-based, no subjective assessor rating influenced the primary trajectory-performance outcomes.

Standard geometric drawing test: The comparison condition consisted of a conventional paper-based Archimedean spiral drawing test. Participants traced a printed Archimedean spiral using a standard pen while their forearm and hand were supported by the drawing surface. The paper spiral was printed at the same scale and orientation as the reference spiral used in the AR condition. The paper-based test was performed under the same lighting conditions and in the same seated position as the AR assessment. Unlike the AR condition, the standard test provided continuous mechanical support through contact with the drawing surface, which served as the principal procedural difference between the two assessment conditions.

### 2.11. Digitization and Scoring of the Standard Paper-Based Test

After completion of the paper-based Archimedean spiral drawing task, each drawing was digitized using a flatbed scanner at 600 dpi resolution under fixed acquisition settings. The scanned images were converted to grayscale and processed automatically. The participant’s spiral trajectory was extracted by image binarization, morphological thinning, and contour tracing. The extracted trajectory was resampled into a sequence of uniformly spaced points in the same normalized coordinate system used for the AR analysis.

The digitized paper trajectory was aligned with the reference Archimedean spiral by translation and uniform scaling so that the center and outer radius matched the reference spiral geometry. After coordinate normalization, the same trajectory-performance scoring algorithm used for the AR condition was applied to the paper-based trajectory. The identical control-point sequence, deviation conditions, penalty coefficients (${const}_{1}$ = 1.0, $p_{const1}$ = 0.7, ${const}_{2}$ = 1.1, $p_{const2}$ = 0.1), acceptance radius, skip-penalty rule, and algorithm-positive threshold (*T* = 70) were used without modification.

Consequently, the comparison between the AR- and paper-based conditions was based on the same trajectory-processing pipeline and the same scoring algorithm, with the principal difference between the two conditions being the presence of continuous mechanical support in the paper-based drawing task and the absence of such support in the AR task.

### 2.12. Participant Characteristics

The available demographic and clinical characteristics of the study participants are summarized in Table 1. Because the present study was designed as a pilot feasibility investigation focused on trajectory-performance assessment, several clinical variables were not systematically collected. To avoid ambiguity, variables that were unavailable are explicitly reported as “not collected”.

Participants with severe upper-limb orthopedic impairment preventing completion of the drawing task, inability to understand task instructions, or inability to complete the experimental procedure were excluded from participation. No additional standardized assessment of Parkinson’s disease severity, medication state, traumatic brain injury severity, blast exposure history, PTSD, or neuropsychiatric comorbidities was performed within the study protocol.

These missing clinical variables represent an important limitation of the study and restrict interpretation of the observed trajectory-performance differences across clinical subgroups.

### 2.13. Statistical Analysis

Because this study was designed as a pilot feasibility study, the statistical analysis was primarily descriptive. Continuous variables were summarized as means ± standard deviations (SDs) or as the median and interquartile range (IQR), depending on data distribution. Group-level trajectory-performance scores were visualized using box plots and individual data points.

For comparisons between groups, nonparametric methods were considered appropriate because of the small sample size of the Parkinson’s disease subgroup. Because the study was designed as a feasibility investigation and participant-level trajectory-performance scores were not archived for complete statistical reconstruction, the statistical analysis was primarily descriptive.

The TBI cohort consisted of combat veterans with a documented history of traumatic brain injury who were recruited during neurological evaluation and rehabilitation. Because standardized TBI severity classification (mild, moderate, severe), Glasgow Coma Scale scores, blast-exposure history, and detailed neuropsychiatric characterization were not systematically collected, the TBI group should be regarded as a heterogeneous clinical cohort.

The primary purpose of the statistical analysis was to describe the distribution of algorithmic trajectory-performance scores across the control, PD, and TBI groups and to estimate the magnitude and uncertainty of the observed group differences. Because the study lacked an independent clinical reference standard and included only six participants with Parkinson’s disease, formal estimates of diagnostic performance (sensitivity, specificity, positive predictive value, negative predictive value, and ROC/AUC) were not considered reliable and were therefore not used to support diagnostic claims. Any observed differences should be interpreted as preliminary and hypothesis-generating.

### 2.14. AR Acquisition and Processing Pipeline

The augmented reality spiral-drawing task was implemented as a real-time vision-based hand-tracking application. The processing pipeline consisted of image acquisition, hand detection, fingertip localization, coordinate transformation, trajectory smoothing, trajectory scoring, and final algorithm-positive classification.

Camera hardware and image acquisition: The system was designed for a standard RGB camera with a minimum resolution of 1280 × 720 pixels operating at 30 frames/s. The camera was positioned approximately 40–60 cm from the participant’s drawing hand and remained fixed throughout each trial. No specialized depth sensor was required.

Software environment: Hand tracking was implemented using MediaPipe Hands (Google MediaPipe, version 0.10.x). The application was developed in Python 3.11 using OpenCV 4.10, NumPy 2.0, and MediaPipe 0.10.x.

Hand landmark model: The MediaPipe Hands landmark model was used for real-time hand-pose estimation. The fingertip trajectory was extracted from the index fingertip landmark (landmark index 8).

Frame-processing pipeline: Each captured RGB frame underwent the following steps:

- RGB image acquisition;
- Hand detection;
- Hand landmark estimation;
- Extraction of landmark 8 coordinates;
- Confidence verification;
- Coordinate transformation to the spiral coordinate system;
- Temporal smoothing;
- Trajectory scoring.

Frames in which no valid hand landmarks were detected were treated as tracking-loss frames.

Coordinate transformation: The detected fingertip coordinates (*u*, *v*) in image space were transformed to the normalized spiral coordinate system by a two-dimensional affine transformation,

$${\left[x,\;y,\;1\right]}^{T}=A\;{\left[u,\;v,\;1\right]}^{T},$$

where *A* is the affine transformation matrix obtained during the application calibration procedure. Transformed coordinates were expressed in the same normalized coordinate system as the reference Archimedean spiral.

Depth handling: The depth coordinate provided by the hand-tracking model was not used in trajectory evaluation. All analyses were performed in the two-dimensional image plane after affine coordinate transformation.

Temporal smoothing: The fingertip trajectory was filtered using a 5-frame moving-average filter to reduce frame-to-frame localization jitter before trajectory scoring.

Hand-detection confidence: Hand landmarks were accepted only when the MediaPipe hand-presence confidence was ≥0.50. Frames below this threshold were rejected.

Tracking-loss handling: A tracking interruption was considered prolonged when valid fingertip detection was absent for more than 15 consecutive frames (approximately 0.5 s at 30 fps). Trials containing prolonged tracking loss were repeated.

Frame exclusion: Across all recorded trials, approximately 3–5% of frames were excluded because of insufficient hand-detection confidence or temporary tracking loss. Excluded frames were not used for trajectory scoring.

Experimental trials: Each participant performed three complete spiral-drawing trials. The trajectory-performance score reported in the analysis corresponded to the best valid trial, defined as the trial without prolonged tracking loss and with the highest trajectory-performance score.

Reference spiral: The displayed reference spiral was an Archimedean spiral with three complete turns. The normalized outer radius was R = 1.0, the spiral scale parameter was a = R/(6π), and the trajectory was sampled into 120 equally spaced control points. The average spacing between adjacent control points was approximately 0.026 normalized units.

Tested hand and handedness: Participants performed the task using their dominant hand. Handedness was recorded by participant self-reporting.

Trial selection and missing-data handling: Each participant performed three complete spiral-drawing trials in both the AR-based and standard paper-based conditions. Before the recorded measurements took place, one familiarization trial was performed and was not included in the analysis.

A trial was considered valid when it was completed without prolonged tracking loss and contained sufficient trajectory data for complete score computation. Prolonged tracking loss was defined as the absence of valid fingertip detection for more than 15 consecutive frames (approximately 0.5 s at 30 frames/s). Trials with prolonged tracking loss were repeated immediately.

For each participant, the best valid trial was selected for analysis. The best trial was defined as the valid trial with the highest trajectory-performance score, thereby minimizing the influence of temporary tracking instability or short-term participant adaptation during repeated testing.

Frames with temporary hand-tracking loss or insufficient detection confidence were excluded from penalty computation. These excluded frames were treated as missing trajectory observations and did not contribute to penalty accumulation. Short interruptions did not invalidate a trial, whereas prolonged tracking loss resulted in trial repetition.

No statistical imputation of missing trajectory points was performed. Missing frames were excluded from trajectory scoring, and only valid trials with complete score computation were included in the final analysis. All participants included in the study completed at least one valid trial, and no participants were excluded from the final analysis because of missing trajectory data.

### 2.15. Ethics Statement

This study was conducted as a non-invasive observational investigation involving an augmented reality spiral-drawing task. The experimental procedure consisted of voluntary recordings of hand movements and did not involve any therapeutic interventions, invasive procedures, administration of medication, or collection of biological samples.

Participation was voluntary, and all participants were informed about the purpose of the study, the recording procedure, and their right to discontinue participation at any time. The collected trajectory data were pseudonymized before analysis, and no names, identification numbers, facial images, or other directly identifiable personal information were retained with the research dataset.

The study was conducted as a pilot methodological feasibility investigation. Informed consent was obtained orally from all adult participants and from the parents or legal guardians of participants younger than 18 years of age prior to participation. All participants and, where applicable, their parents or legal guardians were informed about the purpose and procedures of the study, and participation was voluntary. No directly identifying personal information was collected. The present manuscript reports a retrospective analysis of de-identified trajectory data, and the findings should be considered preliminary and hypothesis-generating. Future validation studies will be conducted prospectively in accordance with applicable institutional ethical requirements and appropriate informed consent procedures.

## 3. Proof-of-Concept Feasibility Evaluation

A total of 131 participants were included in the pilot feasibility study: 50 healthy controls, 6 patients with Parkinson’s disease (PD), and 75 combat veterans with traumatic brain injury (TBI). All participants completed the augmented reality (AR) spiral-drawing task, and the resulting fingertip trajectories were processed using the predefined trajectory-scoring algorithm. The algorithm-positive classification threshold was fixed at T = 70 for all analyses.

### 3.1. Prototype Parameter Evaluation

A preliminary parameter analysis was performed to investigate the influence of the scoring parameters on trajectory tolerance and penalty accumulation. Four parameter configurations were evaluated (see Table 2).

The first configuration demonstrated that the second deviation condition became excessively restrictive, resulting in frequent penalties and unsuccessful completion of the trajectory. Increasing *const*_2_ to 1.3 did not improve the final score when $p_{const2}$ remained high. Reducing p_const2 alone was also insufficient to stabilize the scoring behavior. The fourth configuration (*const*_1_ = 1.0, $p_{const1}$= 0.7, *const*_2_ = 1.1, $p_{const2}$ = 0.1) produced the most stable scoring behavior and allowed successful completion of the trajectory with a final trajectory-performance score of 32/100.

These values represent the prototype configuration selected for the feasibility evaluation and were applied uniformly throughout participant testing.

### 3.2. Group-Level Trajectory-Performance Assessment

The AR-based assessment demonstrated observable differences in algorithmic trajectory behavior between the Parkinson’s disease subgroup and healthy controls (see Table 3). All six participants with Parkinson’s disease were classified as algorithm-positive by the AR-based trajectory assessment. In contrast, the majority of healthy controls were classified as algorithm-negative.

The TBI subgroup demonstrated substantial overlap with the healthy control group. Most participants with TBI completed the AR task successfully and were classified as algorithm-negative, although a limited number of additional algorithm-positive findings were observed in the AR condition.

### 3.3. Comparative Observations

The Parkinson’s disease subgroup consistently demonstrated lower trajectory-performance scores than healthy controls during the AR task. The observed trajectory deviations included reduced trajectory stability, greater accumulation of penalties, and failure to maintain continuous adherence to the reference Archimedean spiral.

**Figure 6:**
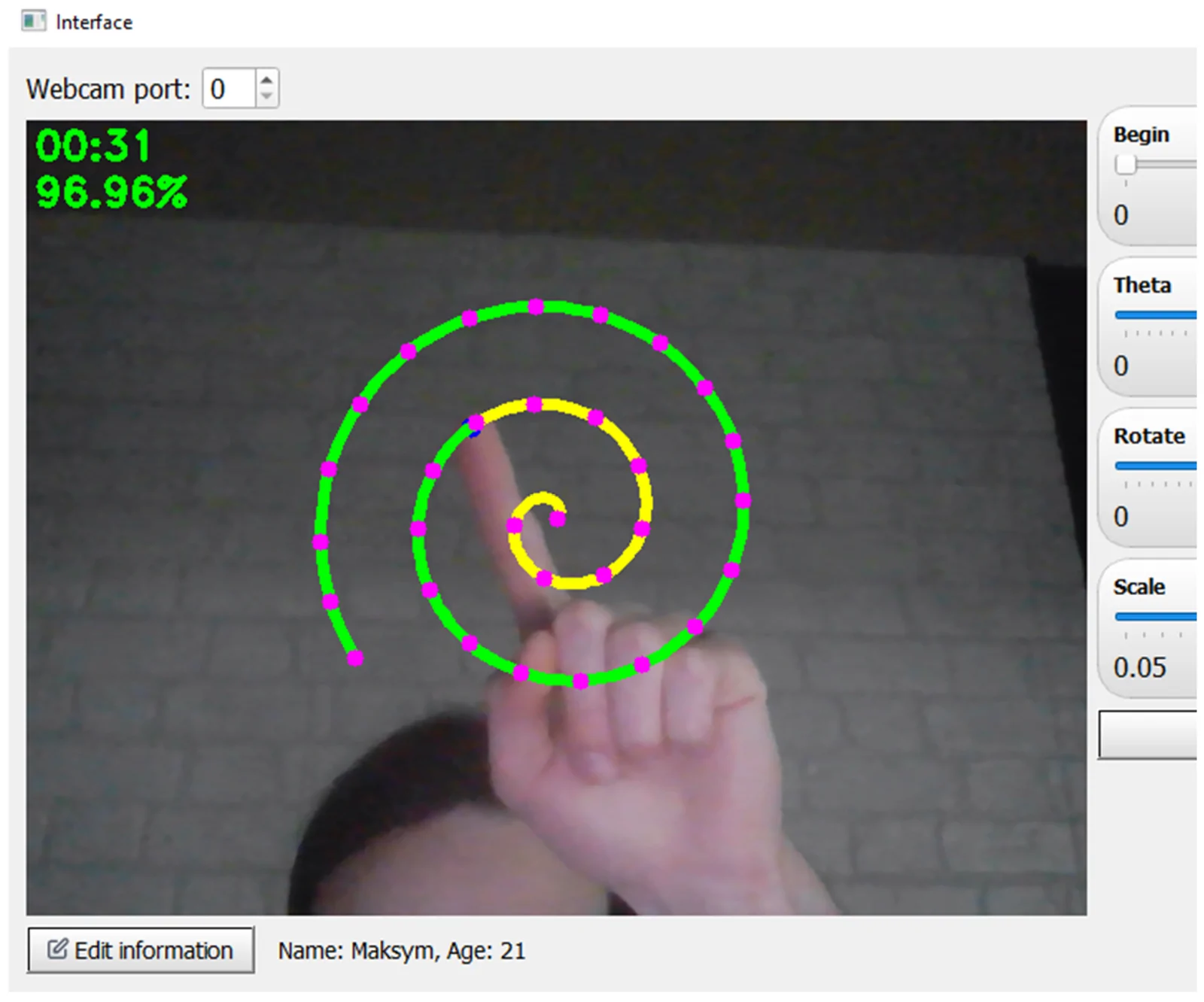
Drawing Archimedes’ reference spiral.

The TBI subgroup showed considerable heterogeneity (Figure 6). Although several participants exhibited noticeable trajectory deviations, the overall distribution of algorithmic scores overlapped substantially with that of the healthy control group. Consequently, in the present pilot cohort, the AR-based assessment distinguished the PD subgroup from healthy controls more clearly than it distinguished the heterogeneous TBI subgroup from healthy controls.

### 3.4. Interpretation of Algorithmic Findings

The AR assessment generated algorithm-positive classifications based exclusively on the predefined trajectory-scoring algorithm. The trajectory-performance score quantified cumulative geometric deviation from the reference spiral under unsupported drawing conditions. Because the study did not include an independent clinical reference standard, the algorithm-positive findings should be interpreted as detections of substantial trajectory deviation rather than as clinically confirmed pathological movement abnormalities.

The prototype experiments demonstrated that the trajectory-performance score was highly sensitive to the penalty parameters and that the selected parameter configuration provided the most stable behavior for the pilot AR-based assessment. The classification of all trajectories from the Parkinson’s disease subgroup as algorithm-positive and the substantial overlap between the TBI and control groups support the feasibility of the proposed anchor-free AR trajectory analysis framework while emphasizing the need for larger clinically validated cohorts and independent diagnostic confirmation.

Given the limited sample size, particularly in the Parkinson’s disease subgroup, these findings should be regarded as preliminary and hypothesis-generating rather than as evidence of diagnostic accuracy or clinical discrimination.

## 4. Discussion I (Discussion of Relevance and Trends)

Let us review the methodological problem: there are currently few, if any, AR studies specifically addressing the screening of military personnel with combat-related TBI for neurodegenerative disorders. Most studies published in Q1 journals support VR/XR/MR technologies for cognitive assessment, rehabilitation, or detection of mTBI consequences in military personnel. In recent years, specialized AR platforms for screening military mTBI have appeared, in particular, Troop READY. Therefore, for the review, it is appropriate to use the broader category of XR (extended reality), which includes AR, MR, and VR, emphasizing that AR is currently at an early stage of development [33]. Accordingly, the purpose of study [33] is to assess the effectiveness, feasibility, and clinical significance of augmented reality (AR) technologies for neurocognitive assessment. In general, this study is a review and fundamentally supports the relevance of our research. Among the most relevant practical studies, the Troop READY (Augmented Reality Return-to-Duty Platform) [34] can be distinguished. This AR-based environment assesses balance, cognitive tasks, motor functions, and situational military scenarios. However, the study does not address neurodegenerative diseases; instead, it examines performance during the execution of certain military tasks with maximum approximation to combat conditions. Its advantage also includes simultaneous assessment of several functional domains and the possibility of field use.

Research is also actively focused on rehabilitation [35], including the VR Cognitive Rehabilitation Protocol for Combat TBI [36]. Immersive scenarios for assessing attention, working memory, and multitasking have been investigated. Limitations: mainly usability was assessed; small sample size; no control group; no analysis of neurodegenerative biomarkers. Another study [37] investigated a VR environment with treadmill walking and cognitive stimuli. Limitations: focus on PTSD rather than TBI screening concept; does not allow for the detection of neurodegenerative changes; requires expensive equipment.

Study [38] investigated the rehabilitation impact of the RehabXR system on patients with chronic vestibular disorders following mild traumatic brain injury (mTBI) at both behavioral and neural levels. The proposed approach employs extended reality (XR) technologies for vestibular assessment and has been shown to influence functional brain connectivity. However, several limitations should be noted: the study primarily focuses on rehabilitation rather than screening, involves a relatively small cohort, lacks long-term monitoring of cognitive decline or dementia, and presents challenges in the interpretation of neuroimaging findings.

Study [39] supports the combined use of diffusion tensor imaging (DTI) and neuropsychological testing for the assessment of traumatic brain injury outcomes. Despite its potential, the approach is constrained by the need for AR/VR technologies and specialized equipment, limited applicability in field settings, high implementation costs, and the inability to perform rapid large-scale population screenings.

Another study [40] combines sensory technologies, motor analysis, and cognitive tests. Limitations: low specificity of individual technologies; lack of an integrated AR platform; significant variability between participants.

Another study [41] describes a 10-year follow-up of motor function after combat mTBI. Limitations: does not use AR; detects sequelae rather than early signs of neurodegeneration; requires long-term follow-up. Specifically, it assesses NfL, GFAP, tau, pTau181, and other biomarkers of neurodegeneration. Limitations: laboratory-based; not suitable for field screening; lacks functional assessment of behavior and cognition; high assay costs.

Another study [42] describes a comprehensive assessment of the effects of blast exposure, PTSD, and mTBI on cognitive function. Limitations: does not use AR; cross-sectional design; difficulty in separating the effects of PTSD from TBI; lack of automated field screening.

Thus, the analysis of contemporary military medicine studies demonstrates that the application of AR/XR technologies in the assessment and management of neurological consequences of combat-related mild traumatic brain injury is a rapidly developing research area. Existing solutions predominantly focus on cognitive rehabilitation, functional assessment, balance evaluation, and monitoring of post-traumatic symptoms, while the use of augmented reality as a dedicated tool for screening and diagnosis of neurodegenerative disorders remains limited.

Most available approaches are characterized by several common limitations, including a primary emphasis on rehabilitation rather than screening, small sample sizes, restricted applicability in field conditions, dependence on expensive and specialized equipment, and insufficient integration of neurodegenerative biomarkers with functional cognitive and behavioral assessments. Furthermore, current studies rarely address the problem of early detection of neurodegenerative changes in military personnel exposed to combat-related mTBI despite the growing evidence linking repetitive brain trauma to long-term neurodegenerative processes.

Therefore, an evident research gap exists regarding the development of integrated, accessible, and cost-effective AR-based diagnostic systems capable of conducting rapid screening for neurodegenerative disorders in military populations under both clinical and field conditions. The creation of such platforms represents a promising and highly relevant direction for future research, combining objective functional assessments, biomarker-based approaches, and the operational advantages of augmented reality technologies.

A further limitation of the present pilot study is that several clinically important participant characteristics were not systematically collected, including standardized PD severity measures and medication states, as well as detailed TBI severity, blast exposure, PTSD, and other neuropsychiatric comorbidities. Consequently, the influence of these factors on AR task performance could not be evaluated.

The lower trajectory-performance scores observed in the AR condition may be related to the absence of a mechanical support surface; however, the present study cannot establish this mechanism conclusively. The AR and standard tests differed in several experimental dimensions, including visual presentation, depth perception, tactile feedback, movement strategy, arm fatigue, and camera-based trajectory acquisition. Consequently, the anchor-free interpretation should be regarded as a hypothesis generated by the pilot observations rather than as a demonstrated causal explanation.

## 5. Discussion II (Research Implications and Prospects)

In this section, we will briefly note that the study was conducted across the participant categories to (1) verify that the concept works; (2) identify unforeseen interferences; and (3) determine how the foreseeable interferences can be mitigated. Evaluation of these three objectives indicated that (1) the system performs data correctly. Therefore, it is potentially possible to take the next step—to explore different classes and try to integrate them. (2.1) This point requires additional interpretation because, in the absence of an anchor (point of support), the system turned out to be much more responsive to trajectory deviations than expected. In particular, the patient (with advanced Parkinson’s disease) was able to draw a standard Archimedean spiral (with classical diagnostic features); however, she was unable to draw the same spiral in augmented reality. We preliminarily conclude that paper or an electronic tablet may serve as an anchor for the brain. By relying on this anchor, the brain partially compensates for the loss of control over the hand (tremor). In augmented reality, this anchor is absent, and therefore, this compensatory support is removed. Currently, the authors are seeking partners from hospital neurology departments for research focused on this problem and on verifying the results. (2.2) Another unexpected result was the phenomenon of the recovery of military personnel (craniocerebral injuries). In particular, the majority of injured military personnel (of different age categories) performed better on the test than the control group. This was observed even in severe cases. Given that more than half of the control group consists of university students, we hypothesize that the stress experienced by civilians living under wartime conditions and the threat of missile attacks may be higher than the stress experienced by military personnel (even with severe injuries and amputations) due to the unpredictability of “tomorrow” (uncertainty). To test this assumption, we are looking for partners to create a control group (healthy participants from a country not involved in armed conflict). (3) The anticipated problems include lighting-related spectral interference. The current mitigation strategy is to avoid direct sunlight and bright light sources behind the participant.

The observed trajectory-performance differences should be interpreted cautiously because psychological, neurological, and rehabilitation-related factors were not systematically assessed in the present study.

## 6. Limitations

This study has several important limitations. First, it was designed as a pilot methodological investigation and included a small Parkinson’s disease cohort, which limits the precision of the observed group differences. Second, the study lacked an independent clinical reference standard for confirmation of algorithm-positive findings. The observed trajectory deviations were not systematically verified by blinded neurological examinations, standardized tremor rating scales, neuroimaging, or independent expert reviews, and therefore the possibility of false-positive findings cannot be excluded.

Third, several clinically important participant characteristics were not systematically collected, including Parkinson’s disease severity, medication state, traumatic brain injury severity, time since injury, blast exposure history, PTSD, rehabilitation status, and other neuropsychiatric comorbidities. Consequently, the influence of these factors on trajectory performance cannot be evaluated.

Fourth, the AR and standard drawing conditions differed in multiple experimental factors, including visual presentation, depth perception, tactile feedback, unsupported arm posture, task novelty, and camera-based trajectory acquisition. Therefore, the present study cannot isolate the specific contribution of the anchor-free condition to the observed trajectory-performance differences.

Finally, the algorithm parameters and the algorithm-positive threshold were selected for prototype feasibility evaluation and were not calibrated against an independent clinical reference standard. The trajectory-performance score should therefore be interpreted as an algorithmic measure of trajectory deviation rather than a clinically validated diagnostic or tremor-severity measure.

Accordingly, the present findings should be interpreted as preliminary evidence of technical feasibility and hypothesis-generating observations rather than evidence of diagnostic accuracy, clinical discrimination, or tremor severity measurement. Future prospective studies with standardized neurological assessments, independent confirmation of algorithm-positive findings, external validation of the scoring algorithm, and prespecified statistical analysis plans will be required to determine the clinical utility of the proposed method.

## 7. Future Work

It should also be noted that, among all participants from the different study groups, there was no case in which a participant was able to see the paper spiral but was unable to perceive the spiral in augmented reality.

This creates two important considerations. First, it raises an accessibility-related issue that should be investigated in future studies. Second, the absence of such cases does not establish the mechanism underlying the observed differences between the AR and standard drawing conditions. The present findings are compatible with the hypothesis that the anchor-free AR environment may influence trajectory performance; however, because the study did not isolate mechanical support from other experimental differences, including visual presentation, depth perception, tactile feedback, unsupported arm posture, task novelty, and camera-based trajectory acquisition, the contribution of anchor removal cannot be determined from the current data. The anchor-free interpretation should therefore be regarded as a hypothesis for future controlled validation studies rather than as a demonstrated explanation of the observed results.

At the beginning of the study, confidence in the feasibility of the method was based primarily on the fundamental difference between the original surface-based “anchor” solution and the proposed “anchor-free” solution. In particular, the system of penalties and control points follows the same general assessment logic as that used in the previously discussed study [19]. Thus, the main difference lies in the method of trajectory acquisition: in the classical approach, a mechanical anchor is present, whereas in the proposed AR-based approach, it is absent. Mathematically, the implementations are different since the classical approach operates in a two-dimensional space and the AR approach operates in a three-dimensional space; however, the evaluation rules are conceptually the same. This provides an objective basis for comparing the two approaches.

The number of familiarization trials required before successful task execution will be included as a predefined secondary outcome in future studies and analyzed using appropriate statistical methods.

If visual perception difficulties occur in a small percentage of participants, this should be considered primarily as an inclusiveness issue. In the present study, although the sample was limited, participants ranged in age from 14 to 82 years, and no case of difficulty perceiving the virtual spiral was observed. There were cases in which participants did not have their glasses, but there was no situation in which a participant could see the paper spiral but could not see the virtual spiral. A larger sample is required for a statistical assessment of this issue; however, the present study is explicitly positioned as a first step aimed at demonstrating the basic functionality of the proposed concept. The next stage of research is planned to include approximately 400–500 participants.

Several issues should be addressed in the next validation stage, as follows.

The sample size was limited, particularly in the Parkinson’s disease group, and the additional trajectory deviation pattern detected in the TBI group involved only a small absolute number of participants. These findings are therefore best interpreted as preliminary and hypothesis-generating. Future studies should include independent confirmation of the AR-detected trajectory deviation pattern using an additional verification method, such as nuclear magnetic resonance imaging (MRI), as was done with the Parkinson’s disease patients. This will allow for better estimation of the false-positive rate and the reporting of standard diagnostic and reliability metrics, including sensitivity.

The current experimental design compares the standard geometric drawing test with the proposed AR protocol as complete procedures. To further isolate the contribution of the anchor-free AR environment, a future controlled study should compare traditional paper-based and AR approaches using the same CNN classifier for both conditions. Performance relative to other diagnostic methods can then be assessed using the paper-based method as the baseline. For this purpose, a statistically sufficient dataset of 400–500 experimental samples needs to be collected.

The current score should be regarded as an algorithmic trajectory-performance score rather than as a clinically calibrated severity index. At this stage, the score is not directly mapped to the UPDRS tremor subscore or to any other clinical severity scale. Establishing such a mapping will require larger cohorts with standardized neurological assessments, clinical severity ratings, and repeated measurements to evaluate test–retest reliability. It should be emphasized that the present study aimed only to demonstrate the functionality of the concept and the method.

The proposed penalty function and parameter settings are preliminary. The constants ${const}_{1}$, $p_{const1}$, ${const}_{2}$, and $p_{const2}$ were used to demonstrate the behavior of the prototype scoring algorithm. These values correspond to the prototype configuration selected after preliminary feasibility testing and were kept constant for all participants throughout the clinical evaluation. Future work could include systematic parameter optimization using grid search, sensitivity analysis, cross-validation, and external validation on larger clinically annotated datasets.

The practical advantage of the proposed approach lies in its potential for widespread availability and preliminary remote assessment without wearable sensors or specialized clinical equipment. To evaluate this potential, future studies should implement a mobile or internet-based version of the protocol, recruit larger and more diverse cohorts, and conduct multicenter validation across independent research groups.

During the research, we focused on ensuring scalability and widespread availability; therefore, we chose a standard integrated camera in a consumer-grade laptop manufactured in 2019 (with a seven-nanometer process technology). Thus, we assume that the cameras on all modern laptops are expected to perform at least as well and support correct system operation. As for lighting, the lighting orientation is implemented based on the standard (freely available on the internet) Python library: if the library’s neural network detects the hand, then the proposed system will work on the received data. We investigated, in fact (at this stage without statistics), various real-world settings. These are classrooms with “healthy students” and teaching rooms with “healthy teachers” of different age groups under different lighting: sunlight under clear and cloudy conditions, indoor artificial lighting, and artificial lighting in a darkened room. Based on the results of this first stage of research, direct sunlight and very bright artificial lighting are serious limitations (if the recognition system fails in a bright place, it is enough to dim the light; outdoor testing in direct sunlight should be avoided). There was no need to conduct a more detailed study since we plan to transfer the system to online mode, where, in parallel with the recruitment of more participants and the creation of a larger database, we will introduce a lighting meter and develop a mechanism for assessing the quality of the camera. To avoid overload, we also plan to conduct a separate study.

We note an interesting secondary observation that we cannot explain at the moment. There is only one point: military personnel with traumatic brain injury, disabilities, etc., completed familiarization in a single trial, while civilian participants (including students, Parkinson’s patients, teachers, and doctors) required at least two familiarization trials. This is our observation for future research. Obviously, the data are averaged, and at this time, we are not making any hypotheses—only a descriptive secondary observation for discussion and hypothesis generation.

Please note that in the experimental part, there are no comparison tables with other methods. This is due to the fact that this work is intended only to demonstrate the feasibility of such screening without requiring an in-person visit to a healthcare facility. The second task was to evaluate the technical assumptions underlying the system’s operation. In particular, the study examined participants’ subjective responses and system performance under different lighting conditions and minor sources of interference.

## 8. Conclusions

This pilot study demonstrates the technical feasibility of an augmented reality-based, anchor-free trajectory performance assessment method for recording deviations from a reference Archimedean spiral trajectory. The proposed system enables real-time acquisition of fingertip trajectories, automated trajectory processing, and quantitative evaluation of trajectory performance under an unsupported drawing condition.

The results indicate that the AR-based assessment can capture trajectory-performance characteristics that differ from those obtained with a conventional surface-based drawing task. The proposed trajectory-scoring algorithm provides a reproducible framework for quantifying cumulative trajectory deviations relative to a predefined spiral reference and generating standardized algorithmic trajectory performance scores.

However, the present study was designed as a pilot methodological investigation and was not intended to establish diagnostic accuracy, clinical discrimination, or tremor severity measurements. The observed algorithm-positive trajectory deviations represent detections generated by the proposed scoring algorithm and should not be interpreted as clinically confirmed trajectory deviations or evidence of diagnostic effectiveness. Similarly, the anchor-free interpretation remains a hypothesis because the current experimental design did not isolate mechanical support from other differences between the AR and standard drawing conditions.

Several important limitations must be considered. The Parkinson’s disease cohort was small; clinical severity and medication states were not systematically characterized; traumatic brain injury severity and time since injury were not consistently recorded; and algorithm-positive findings were not independently confirmed by standardized neurological examination, validated tremor rating scales, neuroimaging, or blinded expert review. In addition, the algorithm parameters and classification threshold require external validation in independent cohorts.

Overall, the proposed AR-based trajectory-performance assessment system should be regarded as a technically feasible platform for quantitative trajectory analysis rather than as a clinically validated diagnostic tool. Future prospective studies with larger participant cohorts, a standardized neurological assessment, validated clinical outcome measures, independent confirmation of algorithm-positive findings, and prespecified statistical analysis plans will be required to determine the reproducibility, clinical validity, and practical utility of the method.

## Figures and Tables

**Table 1:** Participant characteristics by study group.

Characteristic	Healthy Controls (n = 50)	TBI (n = 75)	Parkinson’s Disease (n = 6)
Number of participants	50	75	6
Age, years (range)	14–82	18–55	60–72
Mean age, years	Not systematically recorded	Not systematically recorded	Not systematically recorded
Sex (male/female)	Not systematically recorded	Not systematically recorded	Not systematically recorded
Dominant hand	Self-reported	Self-reported	Self-reported
Hand tested	Dominant hand	Dominant hand	Dominant hand
Parkinson’s disease diagnosis	Not applicable	Not applicable	Clinical diagnosis confirmed by a treating neurologist
TBI history	None reported	Documented history of traumatic brain injury	None reported
TBI severity classification	Not applicable	Not systematically collected	Not applicable
Time since TBI	Not applicable	Not systematically collected	Not applicable
PD duration	Not applicable	Not applicable	Not systematically collected
PD medication state during testing	Not applicable	Not applicable	Not systematically collected
UPDRS or other tremor severity scale	Not applicable	Not applicable	Not systematically collected
Visual impairment affecting testing	Exclusion criteria applied	Exclusion criteria applied	Exclusion criteria applied
Upper-limb orthopedic impairment	Exclusion criteria applied	Exclusion criteria applied	Exclusion criteria applied
Neuropsychiatric comorbidities	Not systematically collected	Not systematically collected	Not systematically collected
PTSD assessment	Not applicable	Not systematically collected	Not applicable
Blast-exposure history	Not applicable	Not systematically collected	Not applicable

**Table 2:** Comparison of the evaluated parameter configurations.

const_1_	$p_{const1}$	const_2_	$p_{const2}$	Finger Trajectory	Final Score
1	0.9	0.9	1	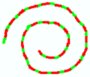	0
1	1	1.3	1	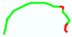	0
1	1	1.3	0.9	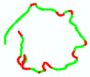	0
1	0.7	1.1	0.1	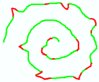	32

**Table 3:** Algorithm-positive classification.

Group	Participants (n)	Algorithm-Positive
Healthy controls	50	Predominantly algorithm-negative
Parkinson’s disease	6	6 (100%)
TBI	75	Predominantly algorithm-negative

## Data Availability

The original contributions presented in this study are included in the article. Further inquiries can be directed to the corresponding authors.
